# Equine allogeneic bone marrow-derived mesenchymal stromal cells elicit antibody responses in vivo

**DOI:** 10.1186/s13287-015-0053-x

**Published:** 2015-04-12

**Authors:** Lynn M Pezzanite, Lisa A Fortier, Douglas F Antczak, Jennifer M Cassano, Margaret M Brosnahan, Donald Miller, Lauren V Schnabel

**Affiliations:** Department of Clinical Sciences, College of Veterinary Medicine, Cornell University, Ithaca, NY 14853 USA; Baker Institute for Animal Health, Cornell University, Ithaca, NY 14853 USA; Department of Clinical Sciences, College of Veterinary Medicine, North Carolina State University, Raleigh, NC 27607 USA

## Abstract

**Introduction:**

This study tested the hypothesis that Major Histocompatibility Complex (MHC) incompatible equine mesenchymal stromal cells (MSCs) would induce cytotoxic antibodies to donor MHC antigens in recipient horses after intradermal injection. No studies to date have explored recipient antibody responses to allogeneic donor MSC transplantation in the horse. This information is critical because the horse is a valuable species for assessing the safety and efficacy of MSC treatment prior to human clinical application.

**Methods:**

Six MHC heterozygote horses were identified as non-ELA-A2 haplotype by microsatellite typing and used as allogeneic MHC-mismatched MSC recipients. MHC homozygote horses of known ELA-A2 haplotype were used as MSC and peripheral blood leukocyte (PBL) donors. One MHC homozygote horse of the ELA-A2 haplotype was the recipient of ELA-A2 donor MSCs as an MHC-matched control. Donor MSCs, which were previously isolated and immunophenotyped, were thawed and culture expanded to achieve between 30x10^6^ and 50x10^6^ cells for intradermal injection into the recipient’s neck. Recipient serum was collected and tested for the presence of anti-donor antibodies prior to MSC injection and every 7 days after MSC injection for the duration of the 8-week study using the standard two-stage lymphocyte microcytotoxicity dye-exclusion test. In addition to anti-ELA-A2 antibodies, recipient serum was examined for the presence of cross-reactive antibodies including anti-ELA-A3 and anti-RBC antibodies.

**Results:**

All MHC-mismatched recipient horses produced anti-ELA-A2 antibodies following injection of ELA-A2 MSCs and developed a wheal at the injection site that persisted for the duration of the experiment. Anti-ELA-A2 antibody responses were varied both in terms of strength and timing. Four recipient horses had high-titered anti-ELA-A2 antibody responses resulting in greater than 80% donor PBL death in the microcytotoxicity assays and one of these horses also developed antibodies that cross-reacted when tested on lymphocyte targets from a horse with an unrelated MHC type.

**Conclusions:**

Allogeneic MSCs are capable of eliciting antibody responses in vivo that can be strong and also cross-reactive with MHC types other than that of the donor. Such responses could limit the effectiveness of repeated allogeneic MSC use in a single horse, and could also result in untoward inflammatory responses in recipients.

**Electronic supplementary material:**

The online version of this article (doi:10.1186/s13287-015-0053-x) contains supplementary material, which is available to authorized users.

## Introduction

The use of autologous mesenchymal stromal/stem cells (MSCs) for the treatment of acute injuries is hindered by the time required to isolate and expand the cells in culture. The genetic background and age of the patient can also affect the quantity and quality of MSCs able to be cultured, making autologous MSC use impossible for some patients regardless of timing issues [1-4]. Banked allogeneic MSCs would be highly advantageous for use in such cases where treatment is indicated at the time of diagnosis or in which it is not feasible to culture quality MSCs from the patient. Controversy exists regarding the immunogenicity of MSCs *in vivo*, despite considerable evidence for their nonimmunogenic and immunosuppressive properties *in vitro* [5-13].

The horse is a valuable species for assessing the safety and efficacy of MSC treatment. Autologous bone marrow-derived MSCs are used routinely in regenerative therapies for equine patients to treat musculoskeletal disorders including tendonitis, osteoarthritis, cartilage damage, and meniscal injuries [14-20]. In addition, the horse allows for noninvasive access to large quantities of samples such as bone marrow aspirate, blood, and serum needed to culture and test the immunogenicity of allogeneic MSCs. It has been shown that equine MSCs are uniformly positive for major histocompatibility complex (MHC) class I expression but are heterogeneous for MHC class II expression [21-24]. MSC MHC class II status is also dynamic in the horse and can vary according to bone marrow aspirate, MSC passage number, and exposure to interferon gamma [24]. When the immunogenicity of MHC-matched or mismatched MSCs obtained from MHC homozygous horses was assessed *in vitro* using modified one-way mixed leukocyte reactions, it was found that MHC class II-positive MSCs caused significantly increased responder T-cell proliferation equivalent to that of the positive control of MHC mismatched peripheral blood leukocytes (PBLs) [24]. As the *in vitro* mixed leukocyte reaction system is used to examine T-cell responses only, no conclusion could be made from this study as to the effect of MHC class I or class II status on the ability of MSCs to elicit a recipient immune response *in vivo*, particularly in terms of antibody production.

Previous studies in swine, rhesus macaques, and mice have shown that, in contrast to their behavior *in vitro*, allogeneic MSCs do elicit cellular and humoral immune responses when implanted in immunocompetent or moderately immunosuppressed animals [25-29]. No studies to date, however, have examined the humoral (antibody) response to allogeneic MSCs in the horse. The purpose of this study, therefore, was to evaluate the immunogenicity of equine MHC-mismatched MSCs *in vivo* using lymphocyte microcytotoxicity assays to measure recipient antibody responses. Our hypothesis was that MHC-mismatched MSCs would elicit recipient antibody responses and that these responses would be independent of MSC MHC class II expression.

## Methods

**Figure 1:**
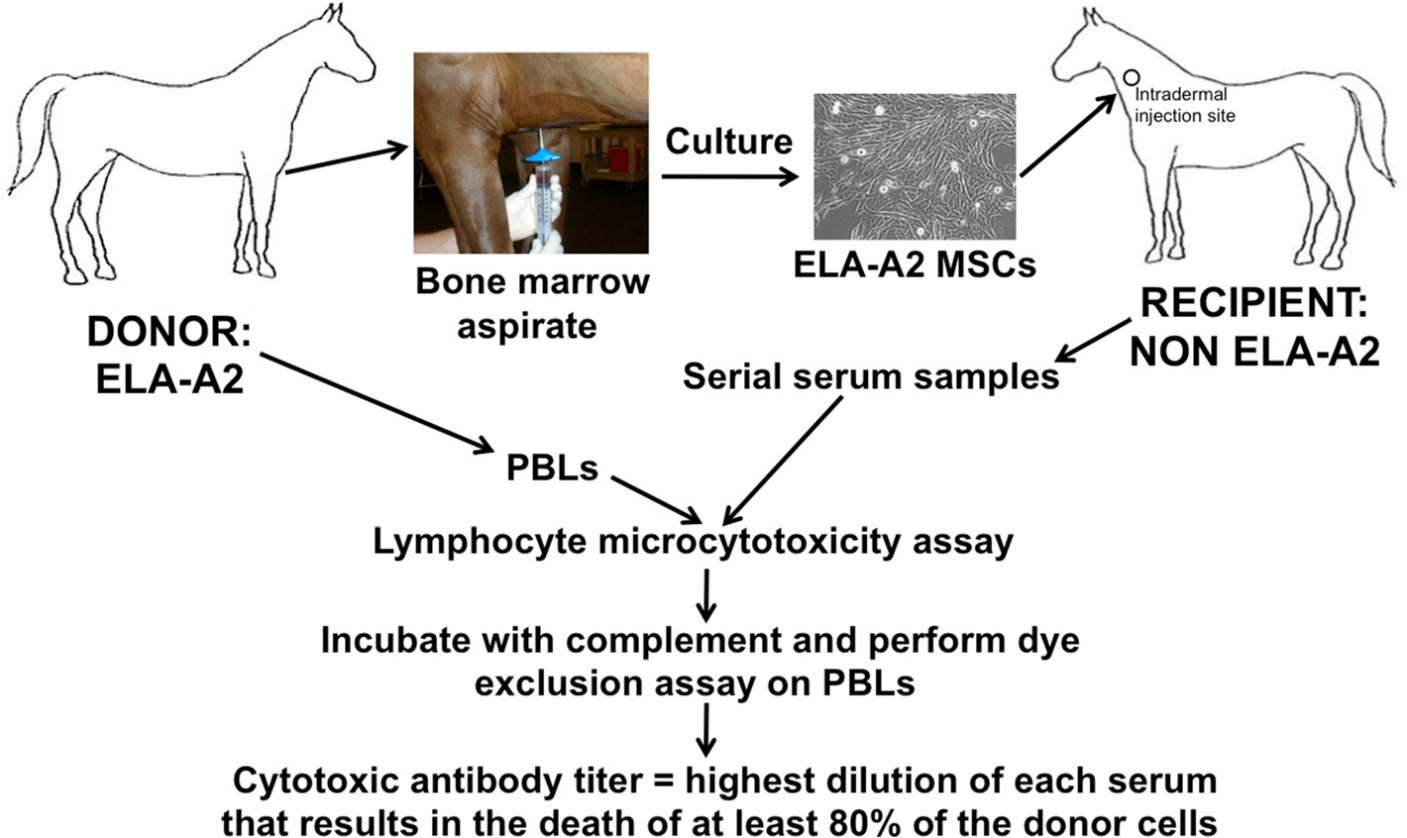
Basic schematic of the study design and methods used. ELA-A2, equine leukocyte antigen haplotype; MSC, mesenchymal stromal/stem cell; PBL, peripheral blood leukocyte.

A schematic of the study design and methods is shown in Figure 1. The Institutional Animal Care and Use Committee of Cornell University approved the use of horses in these studies.

### Donor horse selection

Thoroughbred horses of known MHC haplotype belonging to the Equine Genetics Center at the Baker Institute for Animal Health of Cornell University (Ithaca, New York, USA) were used as MSC and leukocyte donors in these studies. All donor horses were MHC homozygotes of equine leukocyte antigen (ELA) haplotype ELA-A2 as determined previously by ELA serotyping, direct MHC gene sequencing, and microsatellite typing [30-33]. Bone marrow aspirates were collected from these horses and MSCs were isolated and characterized as described previously for use in *in vitro* studies [24]. Specifically, MSCs were immunophenotyped at each passage for expression levels of MHC class I, MHC class II, and a panel of positive (CD44, CD29, CD90) and negative (CD11a/CD18, CD45RB) markers using flow cytometry. Multipotency of the MSCs was also confirmed via trilineage differentiation assays [24]. For consistency, all homozygote horses used in this study (donors and MHC-matched (control) recipient) were identified and referred to by number as established previously (Table 1) [24].

**Table 1:** **Recipient horse and donor MSC information**

***Recipient information***				***Donor (ELA-A2) information***			
**Horse** ^**a**^	**Breed**	**Age (years)**	**Sex**	**Horse** ^**a**^	**MSC passage**	**MSC MHC class II status**	**Number of MSCs injected**
A	Grade	20	Gelding	4 (1)	P4	Negative	46 × 10^6^
B	Thoroughbred	15	Gelding	9	P5	Negative	50 × 10^6^
C	Holsteiner	13	Gelding	ND	P5	Negative	50 × 10^6^
D	Standardbred	16	Gelding	9	P3	Positive	50 × 10^6^
E	Thoroughbred	15	Mare	6	P5	Positive	33 × 10^6^
F	Thoroughbred	18	Gelding	4 (2)	P5	Positive	50 × 10^6^
Control (Horse 2)	Thoroughbred	14	Gelding	9	P5	Negative	50 × 10^6^

ELA-A2, equine leukocyte antigen haplotype; MHC, major histocompatibility complex; MSC, mesenchymal stromal/stem cell; ND, not described. ^a^All MHC-mismatched MSC recipients were identified by letter. All MHC homozygote horses were identified by number including the ELA-A2 homozygote that was the MHC-matched MSC control recipient. MSCs from all donor ELA-A2 homozygotes were immunophenotyped as described previously [24], except for the one donor listed as Horse ND who was not included in the previous study. Horse ND’s MSCs were positive for MHC class I and had an expected MSC marker panel consistent with the other previously described MSCs.

### MHC-mismatched recipient horse selection and pre-injection screening

Six healthy adult horses were used as allogeneic MHC-mismatched MSC recipients in these studies and identified by letters (Horses A to F) (Table 1). These MHC heterozygote horses were identified to be of the non-ELA-A2 haplotype by microsatellite typing using DNA from horses of known serological and microsatellite ELA haplotypes as references (Table 2) [32,34]. All recipient horses were screened and found to be negative for the presence of preexisting anti-ELA-A2 antibodies prior to their use in this study using microcytotoxicity assays as described below.

**Table 2:** **Microsatellite haplotype data for reference horses and MSC recipient horses**

		**Intra-MHC microsatellite alleles**								
		**Class I**		**Class III**	**Class II**					
**Horse**	**Microsatellite haplotype**	**COR110(UMN-JH34-2)**	**TAMU30593**	**ABGe9019**	**ABGe9030**	**TKY3324**	**COR112**	**COR113**	**UM011**	**COR114**
**Reference horse**										
Serological A2/A2	A2	211	343	301	209	269	262	268	174	235
	A2	211	343	301	209	269	262	268	174	235
Serological A3/A3	A3a	207	343	311	211	255	254	260	172	243
	A3a	207	343	311	211	255	254	260	172	243
Serological A3/A3	A3b	207	343	311	211	255	262	268	176	247
	A3c	207	343	311	211	255	262	272	168	255
Serological A5/A5	A5a	221	340	299	212	255	254	260	172	243
	A5a	221	340	299	212	255	254	260	172	243
Serological A9/A9	A9a	217	336	307	215	Null	264	272	168	255
	A9a	217	336	307	215	Null	264	272	168	255
Serological A10/ A10	A10a	221	342	299	207	255	236	266	179	241
	A10b	221	342	311	205	255	236	264	180	243
**Recipient horse**	^a^									
A	A5 like	221	340	299	212	255	254	260	172	243
	Unknown	223	342	297	219	267	262	264	170	239
B	A3 like	207	343	311	211	255	262	260	172	243
	A9a like	217	336	307	215	Null	264	272	168	255
C	Unknown	211	347	299	212	263	254	260	170	239
	A5a like at class II	193	346	318	212	255	254	264	172	243
D	A10a like	221	342	299	Failed	Failed	236	266	179	241
	A10a like	221	340	299	Failed	Failed	236	266	179	241
E	A3b like	207	343	311	211	255	262	268	176	247
	A5a like	221	340	299	212	255	254	260	172	243
F	A10a like	221	342	299	207	Failed	236	266	179	241
	Unknown	193	346	301	211	Failed	264	270	166	249
Control (Horse 2)	A2	211	343	301	209	269	262	268	174	235
	A2	211	343	301	209	269	262	268	174	235

ELA-A2, equine leukocyte antigen haplotype; MSC, mesenchymal stromal/stem cell. ^a^The microsatellite haplotypes assigned to recipient horses in this study were based on knowledge of haplotypes found in other horses in previous studies [29]. Recipient haplotypes are provisional and not definite; however, there was no evidence that any of the recipient horses were compatible with ELA-A2 donors.

### MHC-matched (control) recipient horse selection and pre-injection screening

As a MHC-matched control, one Thoroughbred MHC homozygote horse of the ELA-A2 haplotype (Horse 2) belonging to the Equine Genetics Center at the Baker Institute for Animal Health of Cornell University was the recipient of MSCs from a different ELA-A2 donor. This horse was also screened and found to be negative for the presence of preexisting anti-ELA-A2 antibodies prior to use in this study.

### Preparation and intradermal injection of donor MSCs

Passage 2 MSCs isolated previously from horses of ELA haplotype ELA-A2 and identified as either MHC class II-negative or class II-positive were thawed from liquid nitrogen storage and expanded by *in vitro* culture as described previously until 30 × 10^6^ to 50 × 10^6^ cells were attained (Table 1) [24]. This number of cells was chosen based on a clinically relevant range as well as previous studies in the literature from other species in which the stem cell dose was calculated according to body weight [25-27]. MSCs were lifted from tissue culture plates using Accumax cell dissociation solution (Innovative Cell Technologies Inc., San Diego, CA, USA), washed three times with phosphate-buffered saline to remove residual fetal bovine serum (FBS), counted, and resuspended in 1 ml phosphate-buffered saline for injection. MSCs were maintained at room/ambient temperature during transport from the laboratory to the recipient horse [35]. Immediately prior to MSC injection, an approximately 10 cm × 10 cm area on the left side of the recipient’s neck was clipped and prepared in a sterile manner. The MSC suspension was then injected intradermally using a 20 G needle. Recipient horses were monitored for increased respiratory rate, heart rate, and body temperature as well as swelling, heat, and pain at the site of injection for the duration of the study.

### Recipient serum sampling

For each recipient, a blood sample was collected pre injection, at the time of injection, and every 48 hours for 4 weeks following injection. After 4 weeks, blood samples were taken every 7 days for an additional 4 weeks. At each collection, approximately 10 ml of blood was collected into serum blood collection tubes using a Vacutainer needle (BD Vacutainer, Franklin Lakes, NJ, USA). Serum was allowed to separate for 8 hours at 4°C and the tubes were then centrifuged at 800 × *g* for 10 minutes at 4°C. Approximately 3 ml of serum from each time point was aliquoted and frozen at −20°C for later use in microcytotoxicity assays.

### Donor peripheral blood leukocyte isolation

Blood was collected via jugular venipuncture with extension sets (Baxter Healthcare, Deerfield, IL, USA) and 16 G needles into 250 ml evacuated containers (Baxter Healthcare) each containing 3,750 units of heparin (Sigma-Aldrich, St. Louis, MO, USA). Plasma was allowed to separate for 20 minutes at room temperature and PBLs were then isolated from the plasma via carbonyl iron (Sigma-Aldrich) granulocyte depletion and Ficoll-Paque Plus (Amersham Biosciences, Piscataway, NJ, USA) gradient centrifugation [24]. This PBL isolation technique consistently results in a range of lymphocyte purity between 95 and 99% consisting of 80 to 90% T cells and 10 to 20% B cells [24,36]. Isolated PBLs were resuspended in phosphate-buffered saline and used fresh for all microcytotoxicity assay experiments at a concentration of 30 × 10^6^ cells/ml.

### Microcytotoxicity assays to test for the development of recipient anti-ELA-A2 antibodies

The standard two-stage microcytotoxicity dye-exclusion test was used to detect recipient antibody responses as described previously for transplantation studies in the horse [36,37]. Briefly, fresh PBLs from a donor horse of the haplotype ELA-A2 were tested against serially diluted antisera (neat to 1:2,048) from the MSC recipient. One microliter of diluted antisera and 1 μl donor PBL suspension were incubated for 30 minutes at room temperature under oil in wells of Terasaki plates (Robbins Scientific Corporation, Sunnyvale, CA, USA), following which 5 μl of rabbit complement (Pel-Freez Biologicals, Rogers, AR, USA) was added for an additional 1 hour. The wells were then stained with 2 μl of 5% eosin dye and fixed with 5 μl of 37% formalin (pH between 7.2 and 7.4). The cytotoxic antibody titer was determined by the highest dilution of each antisera that resulted in killing of at least 80% of the donor PBLs [36,37]. All experiments were run in duplicate and donor cell death was assessed by two authors. For each experiment, previously established antibody positive or negative sera were used as internal controls.

### Secondary response study for recipient anti-ELA-A2 antibodies

To determine the secondary response to MHC-mismatched allogeneic MSCs after possible immune priming, any MHC-mismatched recipient horse with a negative or weak primary antibody response was to receive a repeat, or second, injection of donor ELA-A2 MSCs on the right side of the neck at 5 weeks post initial injection.

### Assays to assess the specificity of cytotoxic antibody responses

To test for antibody specificity against the ELA haplotype, the serially diluted antisera (neat to 1:2,048) were tested against fresh PBLs from a donor homozygote horse of the ELA-A3 haplotype (Horse 5 [24]) using the same time points as described above through to 4 weeks and the same methods as described above but with internal control sera either negative or positive for anti-ELA-A3 antibodies. To test for recipient formation of anti-red blood cell lysin and agglutinin antibodies, serum samples from the recipients were submitted to the Hematology Laboratory at the University of California Davis William R. Pritchard Veterinary Medical Teaching Hospital (Davis, California, USA). Samples tested were pre MSC injection, at the peak of anti-ELA-A2 response, and at the end of the study (8 weeks).

## Results

### Recipient physical examination findings

All six MHC-mismatched recipient horses developed a wheal at the injection site that persisted for the duration of the experiment. For both MHC-mismatched recipient horses and the control MHC-matched recipient horse, vital parameters such as respiratory rate, heart rate, and body temperature remained within normal limits for the duration of the study.

### Microcytotoxicity assay performance

Lymphocyte viability after depletion of granulocytes via carbonyl iron and isolation using Ficoll-Paque Plus gradient centrifugation was >95%. Internal control sera known to be positive for antibodies against the donor PBLs resulted in 80 to 100% donor PBL death and known negative sera caused no detectable donor PBL death in all assays performed (Table 3; Additional file 1).

**Table 3:** **Microcytotoxicity assay results for the presence of anti-ELA-A2 antibodies in recipient sera**

		***Recipient serum dilution***											
**Recipient horse**	**Day**	**Neat**	**1:2**	**1:4**	**1:8**	**1:16**	**1:32**	**1:64**	**1:128**	**1:256**	**1:512**	**1:1,024**	**1:2,048**
A	0	0	0	0	0	0	0	0	0	0	0	0	0
A	7	0	0	0	0	0	0	0	0	0	0	0	0
A	14	0	0	0	0	0	0	0	0	0	0	0	0
A	21	0	0	0	<10	<10	<10	0	0	0	0	0	0
A	28	20	30	35	30	30	15	10	10	10	10	10	10
A	35	0	0	0	0	0	0	0	0	0	0	0	0
A	Reinjection = 36	0	0	0	0	0	0	0	0	0	0	0	0
A	42	60	<10	0	0	0	0	0	0	0	0	0	0
A	49	*90*	<10	0	0	0	0	0	0	0	0	0	0
A	56	50	<10	0	0	0	0	0	0	0	0	0	0
B	0	0	0	0	0	0	0	0	0	0	0	0	0
B	7	0	0	0	0	0	0	0	0	0	0	0	0
B	14	80	80	100	90	10	1	15	0	0	0	0	0
B	21	100	100	100	100	*80*	25	35	20	10	10	5	0
B	28	45	75	90	80	*80*	45	15	15	15	25	20	25
B	35	80	90	*100*	50	0	0	0	0	0	0	0	0
B	42	80	*100*	25	0	0	0	0	0	0	0	0	0
B	49	80	*90*	0	0	0	0	0	0	0	0	0	0
B	56	*80*	30	0	0	0	0	0	0	0	0	0	0
C	0	0	0	0	0	0	0	0	0	0	0	0	0
C	7	45	95	80	15	15	1	0	0	0	0	0	0
C	14	100	100	100	100	100	100	100	100	100	100	*80*	30
C	21	100	100	100	100	100	100	100	100	100	100	*85*	50
C	28	100	100	100	100	100	100	100	100	*100*	55	25	15
C	35	100	100	100	100	100	100	100	*100*	0	0	0	0
C	42	100	100	100	100	100	100	*100*	50	0	0	0	0
C	49	100	100	100	100	100	100	*100*	0	0	0	0	0
C	56	100	100	100	100	100	*100*	0	0	0	0	0	0
D	0	0	0	0	0	0	0	0	0	0	0	0	0
D	7	0	0	0	0	0	0	0	0	0	0	0	0
D	14	0	0	0	0	0	0	0	0	0	0	0	0
D	21	0	0	10	25	25	10	0	0	0	0	0	0
D	28	10	10	10	10	10	10	10	10	10	10	10	10
D	35	0	0	0	0	0	0	0	0	0	0	0	0
D	Reinjection = 36	0	0	0	0	0	0	0	0	0	0	0	0
D	42	0	0	0	0	0	0	0	0	0	0	0	0
D	49	0	0	0	0	0	0	0	0	0	0	0	0
D	56	0	0	0	0	0	0	0	0	0	0	0	0
E	0	0	0	0	0	0	0	0	0	0	0	0	0
E	7	0	0	0	0	0	0	0	0		0	0	0
E	14	65	70	*100*	50	30	15	15	15	10	10	10	10
E	21	50	60	*95*	50	15	15	15	15	10	10	10	10
E	28	25	*90*	35	15	10	10	10	10	10	10	10	10
E	35	0	*80*	50	0	0	0	0	0	0	0	0	0
E	42	25	25	0	0	0	0	0	0	0	0	0	0
E	49	25	0	0	0	0	0	0	0	0	0	0	0
E	56	25	0	0	0	0	0	0	0	0	0	0	0
F	0	0	0	0	0	0	0	0	0	0	0	0	0
F	7	0	0	0	0	0	0	0	0	0	0	0	0
F	14	25	25	<10	<10	<10	<10	10	15	20	20	20	20
F	21	90	95	95	100	100	*90*	65	50	25	15	20	10
F	28	40	65	95	90	*80*	25	20	20	15	10	15	15
F	35	40	80	*90*	0	0	0	0	0	0	0	0	0
F	42	24	*80*	25	0	0	0	0	0	0	0	0	0
F	49	40	*90*	10	0	0	0	0	0	0	0	0	0
F	56	20	*80*	25	0	0	0	0	0	0	0	0	0
Control (Horse 2)	0	0	0	0	0	0	0	0	0	0	0	0	0
Control (Horse 2)	7	0	0	0	0	0	0	0	0	0	0	0	0
Control (Horse 2)	14	0	0	0	0	0	0	0	0	0	0	0	0
Control (Horse 2)	21	0	0	0	0	0	0	0	0	0	0	0	0
Control (Horse 2)	28	0	0	0	0	0	0	0	0	0	0	0	0
Control (Horse 2)	35	0	0	0	0	0	0	0	0	0	0	0	0
Control (Horse 2)	42	0	0	0	0	0	0	0	0	0	0	0	0
Control (Horse 2)	49	0	0	0	0	0	0	0	0	0	0	0	0
Control (Horse 2)	56	0	0	0	0	0	0	0	0	0	0	0	0
Internal control (A2 – sera)	Days 0 to 28	0	0	0	0	0	0	0	0	0	0	0	0
Internal control (A2 – sera)	Days 35 to 56	0	0	0	0	0	0	0	0	0	0	0	0
Internal control (A2 + sera)	Days 0 to 28	100	100	100	100	100	100	100	100	100	100	100	100
Internal control (A2 + sera)	Days 35 to 56	100	100	100	100	100	100	100	100	100	100	100	100

**Figure 2:**
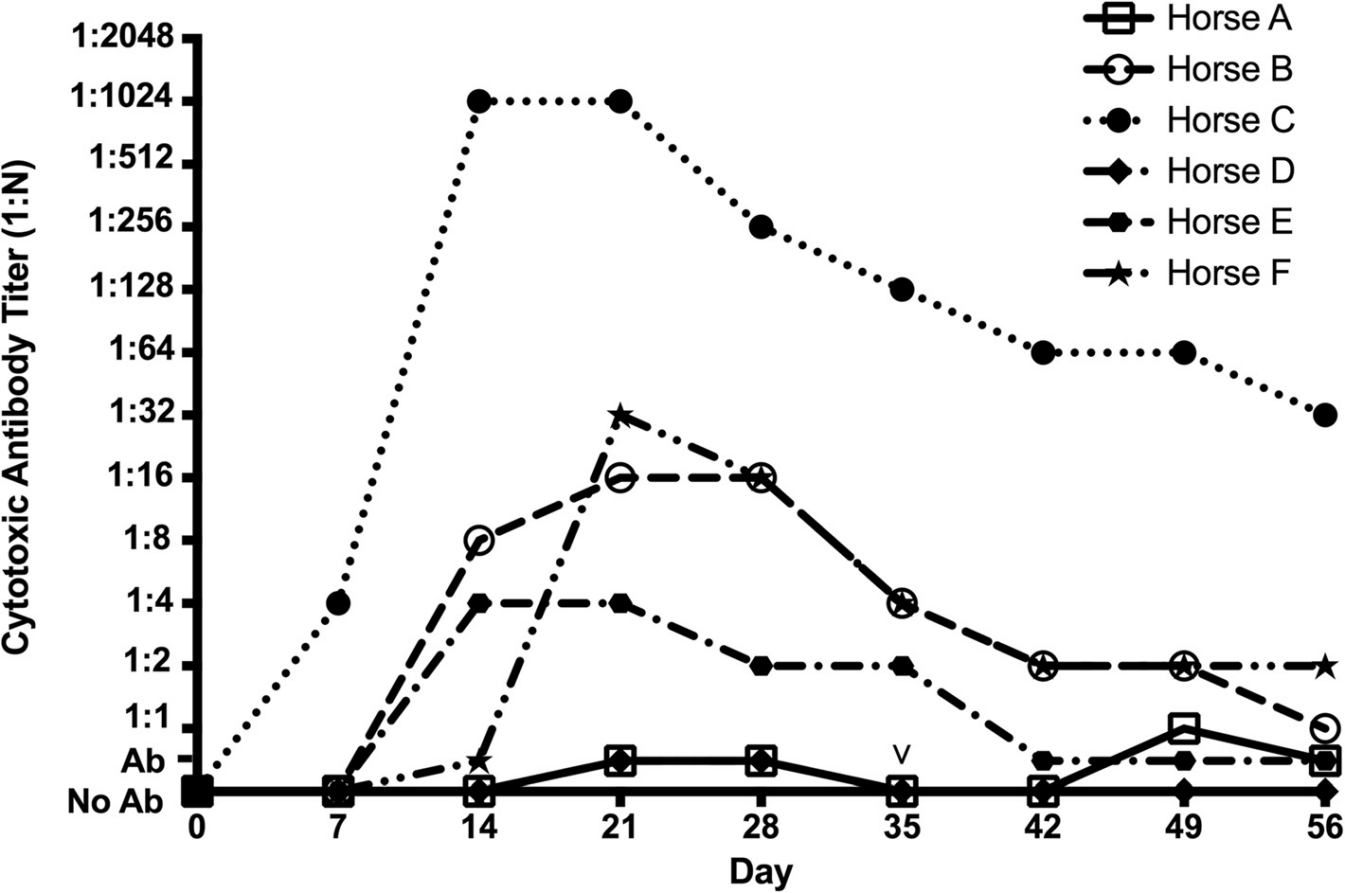
Cytotoxic antibody responses to allogeneic (major histocompatibility complex class I and class II mismatched) donor ELA-A2 mesenchymal stromal/stem cells by recipient horses A to F. The cytotoxic antibody titer was determined by the highest dilution (N) of each recipient serum sample that resulted in killing of at least 80% of donor peripheral blood leukocytes (PBLs). Ab, antibodies detected by the presence of donor PBL death, but with donor PBL death below the cutoff value of 80%; No Ab, no antibodies detected (no donor PBL death). As shown, four recipient horses (Horses B, C, E, and F) had strong anti-ELA-A2 antibody responses which persisted for the duration of the experiment, while two recipient horses (Horses A and D) had weak responses. ^∨^Both weak responders A and D received a second injection of donor mesenchymal stromal/stem cells at 5 weeks (35 days). ELA-A2, equine leukocyte antigen haplotype.

Data reported as the percentage death of ELA-A2 donor leukocytes. For each time point, the value in italics is the cytotoxic antibody titer (the highest dilution of each antisera that resulted in killing of at least 80% of the donor leukocytes as reported in Figure 2). ELA-A2, equine leukocyte antigen haplotype.

### Microcytotoxicity assays to test for the production of anti-ELA-A2 antibodies by MHC-mismatched recipients and by the MHC-matched (control) recipient

The sera from all seven recipient horses were tested in the lymphocyte microcytotoxicity assay against target cells from a single ELA-A2 homozygous horse (Horse 6 in a previous study [24]). All MHC-mismatched recipient horses produced cytotoxic antibodies following intradermal injection of ELA-A2 MSCs (Figure 2), but the control horse did not (Table 3). The cytotoxic antibody responses were varied in strength and timing. Four recipient horses (Horses B, C, E, and F) had strong antibody responses resulting in >80% donor PBL death in the microcytotoxicity assays. These four horses all developed antibodies by 14 days (range 7 to 14 days) and had peak cytotoxic antibody titers ranging from 1:4 (Horse E) to 1:1,024 (Horse C). In addition, all four of these horses had circulating antibodies at the conclusion of the study at 56 days post injection. For Horse C, the cytotoxic antibody titer remained high at 1:64 on day 56.

The remaining two MHC-mismatched recipient horses (Horses A and D) had weak responses resulting in donor PBL death less than the predefined 80% cutoff used in the microcytotoxicity assay for reporting titers. Nevertheless, the antisera from both of these horses resulted in repeatable 10 to 35% donor PBL death at dilutions up to 1:32 on day 21 and up to 1:2,048 on day 28 post injection. The control MHC-matched recipient horse sera never showed any donor cell killing at any dilution at any time point (Table 3).

No correlation was found between MHC class II status of the donor MSCs and recipient antibody response in this study. Of the four recipient horses with strong antibody responses, two received MHC class II-negative MSCs and two received MHC class II-positive MSCs (Table 1). Similarly, no correlation was found between number of donor MSCs injected and the recipient anti-ELA-A2 antibody response. The lowest number of donor MSCs used (33 × 10^6^ cells injected into recipient Horse E) still produced a strong antibody response as compared with those produced by the two weak responders (Horses A and D), who received 46 × 10^6^ and 50 × 10^6^ cells, respectively.

### Secondary response study for the production of anti-ELA-A2 antibodies by MHC-mismatched recipients

Horses A and D with weak anti-ELA-A2 antibody responses following initial ELA-A2 MSC injection received a second injection of ELA-A2 MSCs at week 5 (day 36) of the study (Figure 2 and Table 3). Following this second injection, Horse A did go on to develop anti-ELA-A2 antibodies resulting in >80% donor PBL death in the microcytotoxicity assays with a titer of 1:1 (neat). Horse D, however, did not show any evidence of an anti-ELA-A2 response following the second MSC cell injection (Figure 2 and Table 3).

### Specificity of cytotoxic antibody responses

Only Horse C, the horse with the strongest anti-ELA-A2 response (Figure 2 and Table 3), developed anti-ELA-A3 antibodies post injection resulting in ELA-A3 donor PBL death (Additional file 1). These results indicate that the cytotoxic antibodies produced as a result of MSC injection were directed against epitopes of the MHC class I antigens of the ELA-A2 homozygous donor, because there is no detectable sharing of the MHC class I antigens themselves between the ELA-A2 and ELA-A3 haplotypes [33]. Cross-reactivity of high-titered anti-MHC antisera generated in transplantation responses is well known [38].

Similarly, Horse C was the only recipient to develop unidentified anti-red blood cell lysin antibodies, which were present in his serum at the peak of his anti-ELA-A2 response. Horse D, who had a weak primary anti-ELA-A2 response and did not show any evidence of an increased anti-ELA-A2 response following a repeat ELA-A2 MSC injection, did develop unidentified anti-red blood cell agglutinin antibodies following the repeat MSC injection (data not shown).

## Discussion

The results of this study demonstrate that allogeneic equine bone marrow-derived MSCs can elicit strong antibody responses after intradermal injection into MHC-mismatched recipients. Although the strength of the responses varied between recipients, all recipients developed antibodies against lymphocytes of the donor ELA-A2 haplotype. Four of the recipient horses generated strong responses in which antibodies were present by 7 to 14 days and persisted for the duration of the study. Overall, the responses seen in these horses were very similar to those reported previously in horses following ectopic trophoblast allotransplantation [37]. Skin allografting and allogeneic pregnancy in the horse can also induce cytotoxic antibodies with similar response profiles [39]. Antibody responses to allogeneic MSC transplantation have been reported previously for other species [25-27]. These results in the horse add to the growing body of evidence that MSCs are not immunoprivileged, as originally believed, and suggest that caution must be exercised when considering the use of allogeneic MSCs.

It is important to note that although FBS was used in the MSC culture media in this study, FBS cannot be the target of the anti-donor antibody responses observed. The microcytotoxicity assays used are extremely specific for antibodies in the recipient antisera that are targeting and killing donor PBLs. As the donor PBLs do not express bovine proteins and are never in contact with FBS during their isolation or culture within the assay, anti-FBS antibodies cannot be responsible for their death. While the use of FBS in MSC culture media up until the time of injection is certainly a potential concern for clinical application and should be avoided in a clinical setting or in future studies examining donor MSC viability *in vivo* [40], FBS was useful in this study to standardize donor MSC culture conditions and growth. Such standardization would have been impossible using autologous serum from each donor.

There are several conceivable reasons for the variation in the strength of the antibody responses observed in this study. The first is potential differences in recipient health and immune status. Although all recipient horses had normal physical examinations and were in fair to good body condition, it is interesting to note that the two weak responders (Horses A and D) were the only two recipient horses recently added to the research herd, while the four strong responders were existing members of the university teaching or research herd. It is possible that the immune systems of the two weak responders were not as strong as the other recipients due to factors such as improper nutrition prior to their arrival at the university and/or stress from having gone through the auction and associated transport as well as being placed with new herd members. Such factors could increase endogenous glucocorticoid levels and thereby cause immune suppression [41-43]. As summarized in Table 1, Horse A received MSCs from the same donor horse (Horse 4) as Horse F, who had a strong antibody response. Similarly, Horse D received MSCs from the same donor horse (Horse 9) as Horse B, who had a strong antibody response, suggesting that the recipient health and immune status is the factor likely to be affecting the antibody response.

There is also the possibility that some of the strong responders may have had a previous primary response against the ELA-A2 haplotype. While this seems unlikely given that the majority of these horses had long-term known histories and health records with the university, it is possible that the recipient mare could have been pregnant in the past or that the geldings could have been exposed to ELA-A2 antigens through another mechanism. In particular, recipient Horse C had a response that is more typical of a secondary, or primed, immune response than of a primary immune response. This horse developed detectable antibodies by 7 days post injection and had very high titers that remained high for the duration of the study. While all recipient horses were screened for the presence of the circulating anti-ELA A2 antibodies prior to MSC injection, there was no practical way to screen them for past exposure prior to the MSC injections performed in this study.

Although the authors are of the opinion that the recipients themselves are the likely cause of the observed variability in antibody responses, one must also consider the fact that different MSCs were used. While this was done intentionally to test for possible differences in response to MSC MHC class II expression, this difference could have affected the results in several ways because MSCs are not only heterogeneous in their basal MHC class II expression, but also dynamic in their MHC expression as has been demonstrated previously with interferon gamma stimulation [24]. It is possible that the preinjection expression levels of either MHC class I or MHC class II could have changed post injection. While no differences were observed in this study between responses elicited by MHC class II-positive MSCs compared with MHC class II-negative MSCs, a relatively small number of horses were examined and this study primarily evaluated a MHC class I-driven antibody response. Injection site biopsies to examine a cellular response were considered but ultimately not performed due to concerns about removing the MSC antigen source and thereby altering the antibody response results.

Lastly, the observed results for the two weak responders that received repeat MSC injections might be misleading due to the fact that both horses had circulating anti-ELA-A2 antibodies at least very close to the time of the repeat injection. It is possible that the donor MSCs injected in week 5 may have been targeted by circulating antibodies and destroyed before they could induce a secondary antibody response. This would be a potential explanation for why Horse D did not develop a further antibody response.

The doses of MSCs used in this study were clinically relevant and proportionally similar on a body weight scale (approximately 0.1 million MSCs/kg body weight) to those originally used in allogeneic MSC studies in other species [25-27]. Recent publications from human autologous and allogeneic bone marrow-derived MSC clinical trials, however, have reported using much higher doses ranging from 0.3 to 10 million MSCs/kg body weight, with some evidence that 2 to 5 million MSCs/kg body weight is optimal for immunomodulation [44]. In this study a wide range of MSC doses was not examined. Ultimately, it may not even be feasible to culture enough equine MSCs to reach the higher doses reported for humans on a million MSCs/kg body weight basis.

In this study only bone marrow-derived MSCs were examined and they were delivered via intradermal injection. In addition, semi-allogeneic recipients were not included. The effect that more clinically relevant injection site locations may have on recipient antibody response remains to be determined, as does the effect of the MHC haplotypes of the clinical MSC donor and recipient. Clinical patients are not expected to be MHC homozygotes or completely matched in terms of their MHC haplotype. Given the frequency distribution of MHC haplotypes in breeds such as the Thoroughbred or Standardbred, it is possible that some recipients would be semi-allogeneic to randomly selected donor MSCs [30-33]. Future studies taking these factors into account and also comparing bone marrow-derived MSCs with other MSC types are certainly warranted.

Finally, perhaps the most important finding of this study in terms of clinical application is the potential for the development of strong, long-lasting, and possibly cross-reactive antibody responses after MSC transplantation. Cross-reactivity with diverse MHC class I antigens happens over time as the immune response progresses and the recipient develops broader and stronger antibodies against other MHC epitopes that may be shared against other MHC haplotypes [38]. While a single dose of allogeneic MSCs might cause no overt harm to the recipient at the time of that injection, there is the possibility for a more severe systemic reaction following a second injection or if the patient was ever to receive other foreign tissues or cells, including a blood transfusion. Also of consideration is the fact that recipients with strong immune responses or with primed immune responses may have enough circulating antibodies and immune effector cells to cause donor MSC death before they have had the chance to exert their desired regenerative effects. Further research is warranted to determine whether MHC class I and II expression levels on MSCs can be manipulated *in vitro* to an extent that the MSCs are no longer immunogenic *in vivo*.

## Conclusions

This study is the first to investigate the antibody response to allogeneic MSC transplantation in the horse. Using known MHC-mismatched donors and recipients, we have shown that allogeneic MSCs are indeed capable of eliciting antibody responses *in vivo*, which can be strong and also cross-reactive with MHC haplotypes other than the donor type. These results highlight the potential risk of clinical allogeneic MSC application.

## Additional file

Additional file 1:
**Is a table presenting microcytoxicity assay results for the presence of anti-ELA-A3 antibodies in recipient sera.** Data are reported as the percentage death of ELA-A3 donor leukocytes.

## Abbreviations

ELA: equine leukocyte antigen
FBS: fetal bovine serum
MHC: major histocompatibility complex
MSC: mesenchymal stromal/stem cell
PBL: peripheral blood leukocyte

## References

[1] Majors AK, Boehm CA, Nitto H, Midura RJ, Muschler GF (1997). Characterization of human bone marrow stromal cells with respect to osteoblastic differentiation. J Orthop Res.

[2] Peister A, Mellad JA, Larson BL, Hall BM, Gibson LF, Prockop DJ (2004). Adult stem cells from bone marrow (MSCs) isolated from different strains of inbred mice vary in surface epitopes, rates of proliferation, and differentiation potential. Blood.

[3] Baxter MA, Wynn RF, Jowitt SN, Wraith JE, Fairbairn LJ, Bellantuono I (2004). Study of telomere length reveals rapid aging of human marrow stromal cells following in vitro expansion. Stem Cells.

[4] Carter-Arnold JL, Neilsen NL, Amelse LL, Odoi A, Dhar MS (2014). In vitro analysis of equine, bone marrow-derived mesenchymal stem cells demonstrates differences within age- and gender-matched horses. Equine Vet J.

[5] Griffin MD, Ryan AE, Alagesan S, Lohan P, Treacy O, Ritter T (2013). Anti-donor immune responses elicited by allogeneic mesenchymal stem cells: what have we learned so far?. Immunol Cell Biol.

[6] Stagg J, Galipeau J (2007). Immune plasticity of bone marrow-derived mesenchymal stromal cells. Handb Exp Pharmacol.

[7] Griffin MD, Ritter T, Mahon BP (2010). Immunological aspects of allogeneic mesenchymal stem cell therapies. Hum Gene Ther.

[8] Stagg J (2007). Immune regulation by mesenchymal stem cells: two sides to the coin. Tissue Antigens.

[9] Tse WT, Pendleton JD, Beyer WM, Egalka MC, Guinan EC (2003). Suppression of allogeneic T-cell proliferation by human marrow stromal cells: implications in transplantation. Transplantation.

[10] Pittenger MF, Mackay AM, Beck SC, Jaiswal RK, Douglas R, Mosca JD (1999). Multilineage potential of adult human mesenchymal stem cells. Science.

[11] Di Nicola M, Carlo-Stella C, Magni M, Milanesi M, Longoni PD, Matteucci P (2002). Human bone marrow stromal cells suppress T-lymphocyte proliferation induced by cellular or nonspecific mitogenic stimuli. Blood.

[12] Le Blanc K, Mougiakakos D (2012). Multipotent mesenchymal stromal cells and the innate immune system. Nat Rev Immunol.

[13] Le Blanc K, Tammik L, Sundberg B, Haynesworth SE, Ringden O (2003). Mesenchymal stem cells inhibit and stimulate mixed lymphocyte cultures and mitogenic responses independently of the major histocompatibility complex. Scand J Immunol.

[14] Schnabel LV, Fortier LA, McIlwraith CW, Nobert KM (2013). Therapeutic use of stem cells in horses: which type, how, and when?. Vet J.

[15] Schnabel LV, Lynch ME, van der Meulen MC, Yeager AE, Kornatowski MA, Nixon AJ (2009). Mesenchymal stem cells and insulin-like growth factor-I gene-enhanced mesenchymal stem cells improve structural aspects of healing in equine flexor digitorum superficialis tendons. J Orthop Res.

[16] Fortier LA (2012). Making progress in the what, when and where of regenerative medicine for our equine patients. Equine Vet J.

[17] Fortier LA, Travis AJ (2011). Stem cells in veterinary medicine. Stem Cell Res Ther.

[18] Frisbie DD, Smith RK (2010). Clinical update on the use of mesenchymal stem cells in equine orthopaedics. Equine Vet J.

[19] Frisbie DD, Stewart MC (2011). Cell-based therapies for equine joint disease. Vet Clin North Am Equine Pract.

[20] De Schauwer C, Van de Walle GR, Van Soom A, Meyer E (2013). Mesenchymal stem cell therapy in horses: useful beyond orthopedic injuries?. Vet Q.

[21] Guest DJ, Ousey JC, Smith MRW (2008). Defining the expression of marker genes in equine mesenchymal stromal cells. Stem Cell Cloning.

[22] Carrade DD, Lame MW, Kent MS, Clark KC, Walker NJ, Borjesson DL (2012). Comparative analysis of the immunomodulatory properties of equine adult-derived mesenchymal stem cells. Cell Med.

[23] De Schauwer C, Meyer E, Van de Walle GR, Van Soom A (2011). Markers of stemness in equine mesenchymal stem cells: a plea for uniformity. Theriogenology.

[24] Schnabel LV, Pezzanite LM, Antczak DF, Felippe MJ, Fortier LA (2014). Equine bone marrow-derived mesenchymal stromal cells are heterogeneous in MHC class II expression and capable of inciting an immune response in vitro. Stem Cell Res Ther.

[25] Poncelet AJ, Vercruysse J, Saliez A, Gianello P (2007). Although pig allogeneic mesenchymal stem cells are not immunogenic in vitro, intracardiac injection elicits an immune response in vivo. Transplantation.

[26] Isakova IA, Lanclos C, Bruhn J, Kuroda MJ, Baker KC, Krishnappa V (2014). Allo-reactivity of mesenchymal stem cells in rhesus macaques is dose and haplotype dependent and limits durable cell engraftment in vivo. PLoS One.

[27] Badillo AT, Beggs KJ, Javazon EH, Tebbets JC, Flake AW (2007). Murine bone marrow stromal progenitor cells elicit an in vivo cellular and humoral alloimmune response. Biol Blood Marrow Transplant.

[28] Inoue S, Popp FC, Koehl GE, Piso P, Schlitt HJ, Geissler EK (2006). Immunomodulatory effects of mesenchymal stem cells in a rat organ transplant model. Transplantation.

[29] Nauta AJ, Westerhuis G, Kruisselbrink AB, Lurvink EG, Willemze R, Fibbe WE (2006). Donor-derived mesenchymal stem cells are immunogenic in an allogeneic host and stimulate donor graft rejection in a nonmyeloablative setting. Blood.

[30] Lazary S, Antczak DF, Bailey E, Bell TK, Bernoco D, Byrns G (1988). Joint Report of the Fifth International Workshop on Lymphocyte Alloantigens of the Horse, Baton Rouge, Louisiana, 31 October-1 November 1987. Anim Genet.

[31] Tallmadge RL, Lear TL, Antczak DF (2005). Genomic characterization of MHC class I genes of the horse. Immunogenetics.

[32] Tseng CT, Miller D, Cassano J, Bailey E, Antczak DF (2010). Identification of equine major histocompatibility complex haplotypes using polymorphic microsatellites. Anim Genet.

[33] Tallmadge RL, Campbell JA, Miller DC, Antczak DF (2010). Analysis of MHC class I genes across horse MHC haplotypes. Immunogenetics.

[34] Brinkmeyer-Langford CL, Cai JJ, Gill CA, Skow LC (2013). Microsatellite variation in the equine MHC. Anim Genet.

[35] Bronzini I, Patruno M, Iacopetti I, Martinello T (2012). Influence of temperature, time and different media on mesenchymal stromal cells shipped for clinical application. Vet J.

[36] Antczak DF, Bright SM, Remick LH, Bauman BE (1982). Lymphocyte alloantigens of the horse. I. Serologic and genetic studies. Tissue Antigens.

[37] Adams AP, Antczak DF (2001). Ectopic transplantation of equine invasive trophoblast. Biol Reprod.

[38] Sernee MF, Ploegh HL, Schust DJ (1998). Why certain antibodies cross-react with HLA-A and HLA-G: epitope mapping of two common MHC class I reagents. Mol Immunol.

[39] Adams AP, Oriol JG, Campbell RE, Oppenheim YC, Allen WR, Antczak DF (2007). The effect of skin allografting on the equine endometrial cup reaction. Theriogenology.

[40] Horwitz EM, Gordon PL, Koo WK, Marx JC, Neel MD, McNall RY (2002). Isolated allogeneic bone marrow-derived mesenchymal cells engraft and stimulate growth in children with osteogenesis imperfecta: Implications for cell therapy of bone. Proc Natl Acad Sci U S A.

[41] Franchimont D (2004). Overview of the actions of glucocorticoids on the immune response: a good model to characterize new pathways of immunosuppression for new treatment strategies. Ann N Y Acad Sci.

[42] Elenkov IJ, Webster EL, Torpy DJ, Chrousos GP (1999). Stress, corticotropin-releasing hormone, glucocorticoids, and the immune/inflammatory response: acute and chronic effects. Ann N Y Acad Sci.

[43] Elenkov IJ, Chrousos GP (1999). Stress Hormones, Th1/Th2 patterns, Pro/Anti-inflammatory Cytokines and Susceptibility to Disease. Trends Endocrinol Metab.

[44] Sharma RR, Pollock K, Hubel A, McKenna D (2014). Mesenchymal stem or stromal cells: a review of clinical applications and manufacturing practices. Transfusion.

